# Supplementary material for: Are carbon emissions trading and green financial instruments synergistic? -Comprehensive quantitative research based on content analysis
Source: PLoS One. 2024 Mar 7;19(3):e0298601. doi: 10.1371/journal.pone.0298601 (PMC10919601; doi:10.1371/journal.pone.0298601)
Supplement: S1 Data — (ZIP) [file pone.0298601.s001.zip › Data/地级市Z_F工作报告.zip!/░▓╗╒/│n╓▌╩╨/└┤╫╘╣1⁄2╓┌║┼í╛┬φ┐╦╩2╛▌═°í┐_2014.docx]

各位代表：

现在，我代表市人民政府，向大会作工作报告，请予审议，并请市政协委员和其他列席人员提出意见。

一、2013年工作回顾

2013年是我们奋力攻坚、着力转型、全力冲刺的一年。面对国际环境错综复杂、国内经济下行压力加大的严峻形势，全市上下紧紧围绕主题主线，以十大工程、百亿项目、千亿产业为抓手，以“三个一千”活动为保障，坚持“五位一体”，突出“四化同步”，力求稳中求进、稳中有为、稳中提质，实现了经济社会持续快速健康发展。全年完成地区生产总值1086.1亿元，增长11.1%；规模以上工业增加值490.9亿元，增长15.5%；固定资产投资1075.8亿元，增长21.9%；财政收入178.9亿元，增长16.7%；社会消费品零售总额338.5亿元，增长14.4%；外贸进出口18.6亿美元，增长23.8%；城镇居民人均可支配收入22591元，农民人均纯收入9235元，分别增长10.6%和13.5%。突出表现在：

**多项工作取得突破。**GDP和固定资产投资首超千亿，站稳全省第一方阵；规上工业增加值、财政收入分别前移3位、1位，均居全省第六，实现可喜跨越。新一轮城市总规获省规委会通过，正式成为长三角协调会和南京都市圈发展联盟会员城市，整体加入合肥经济圈，纳入长江中游城市群、淮河生态经济带规划区域，定远、明光终于挤入皖北政策扶持范围，为滁州争得新的发展空间、政策空间。华塑一期项目成功投产、二期项目顺利开工。国家园林城市、道路交通二等管理模范城市争创成功，省级文明城市通过考核。小岗村“两年大变样”任务提前完成，成功承办第13届全国村长论坛。

**多个方面获得丰收。**粮食生产实现“八连丰”，总产83亿斤；规上工业总产值逼近2000亿元；财政全面增收，市本级超50亿元，天长超30亿元，各县市区均超10亿元；全年域内银行新增贷款186亿元，增量全省第四，存贷比首超70%，域外银行贷款34亿元，直接融资13.6亿元；城乡居民人均收入高于全国平均水平；滁州海关开关首年入库税款1.55亿元。

**多数地方竞相出彩。**刘延东副总理视察我市基层医改和职教工作，给予充分肯定；张宝顺书记用“热气腾腾、蒸蒸日上、欣欣向荣”形容滁州发展势头；王学军省长对我市项目建设给予高度评价；山西省、苏州市等多个高规格党政代表团来滁考察；实现全国科技进步先进市“两连冠”、全省双拥模范城“七连冠”；荣获2013中国安居宜居价值城市称号；H7N9防控、群众工作体制机制创新、地震应急、群众体育、法律援助、安全生产月活动等工作受到国家部委表彰；校车安全“滁州模式”全国推广，教育信息化建设全国试点；全国“春风行动”启动仪式在我市举行；全省开发区产城一体化规划建设、农田水利基本建设暨造林整地、节约集约用地等多项工作现场会在我市召开；天长被评为全国生态文明先进县、第二届国土资源节约集约模范县、首批国家餐饮服务食品安全示范县，来安被评为全国生态文明先进县、法制创建活动先进县，明光被评为国家卫生应急综合示范县，市开发区获批国家新型工业化产业示范基地和农业产业化示范基地；市直多个部门在全省综合考评中名列前茅。

**多类指标争先进位。**规上工业增加值增速前移4位，居全省第一；第三产业增加值增速前移5位，居全省第五；实际利用外商直接投资增速前移1位，居全省第一。水利建设投资、森林增长面积、粮食收购量、家庭农场总数等，均居全省第一；高速公路建设规模、在建里程、总投资三项指标和国省干线公路建设开工率，均居全省第一。

一年来，我们主要做了以下工作：

**（一）突出“三个一千”，服务大局展现新作为。**

坚持用心、用情、用智，扎实开展“千名干部帮招千个项目、千名干部帮抓千项工程、千名干部帮扶千家企业”活动，领导带头，各尽其责，及时有效帮助各类企业、项目解决落地、建设、生产、经营中的问题上万个。与此同时，按照一月一主题，月月有重点，整合力量，集成攻关，先后开展了招商引资攻坚月、工程建设提速月、经济运行服务月、项目前期工作联动推进月、央企合作落实行动月、百日会战等，力促项目快签约、快建设、快投产。通过努力，用地上，争取建设用地指标6.5万亩，供应6万亩；用工上，举办各类招聘会近500场，帮招员工近9万人次；用电上，加快电网建设，强化用电调度，全市工业用电量60.6亿千瓦时，增长14.1%。

**（二）突出提质提效，项目建设取得新成绩。**

坚持经济工作项目化、项目工作责任化，把抓发展、调结构、惠民生落实到项目上。**谋划争取一批。**围绕转型升级、新型城镇化等方面，谋划亿元以上项目615个，总投资3538亿元；争取国家和省资金21.4亿元。**大力招引一批。**新引进市外亿元以上项目330个，到位资金592亿元，其中南大光电、朝野电视等30亿元以上7个，拉芳日化、德威新材料等10亿元以上40个。**加快实施一批。**新开工规模以上项目1620个，竣工1200个。总投资25亿元的长电科技、23亿元的雀巢银鹭等一批项目竣工投产，总投资80亿元的远东电缆、50亿元的亚太科技五金机电城、30亿元的德轮轮胎、29亿元的长丰扬子等一批项目加快建设。

**（三）突出抓大活小，工业经济跃上新台阶。**

坚持顶天立地、铺天盖地两手抓，提升工业经济综合竞争力。**打造支柱产业。**先进装备、智能家电等六大支柱产业实现产值1580亿元，增长17％，拉动规上工业增长13.9个百分点。战略性新兴产业企业达201家，居全省第三。高新技术企业达111家，居全省第六；实现产值850亿元，居全省第三。**培育龙头企业。**推进“510”计划，狠抓“千亿技改”，共实施500万元以上技改项目315项，完成投资230亿元。加快推进“三上”工程，规模企业达1124户，净增239户。其中，产值亿元以上企业420户，新增35户。**推进全民创业。**开展全民创业提升年活动，新增私营企业5000户、个体工商户1.7万户。16家企业进入省民企百强，居全省第四。**加快技术创新。**获批各级技术中心37家，累计达171家，省级技术中心数全省第三。全柴获批国家级技术中心，全柴动力消失模铸造等3个项目列为国家重大技术装备重点项目。专利申请量和授权量居全省第四和第五。**夯实园区平台。**15个省级以上开发园区投入资金65亿元，新增开发面积25平方公里，总面积达220平方公里，入园企业总数2800多家。市开发区入园企业超千家，全面完成“3344”目标任务。苏滁现代产业园一期12平方公里路网基本建成，入园项目30个，总投资11亿元的新盛诺、10亿元的胜华波等项目加快建设。

**（四）突出规模特色，现代农业迈出新步伐。**

坚持农业基础地位不动摇，加快农业现代化进程。**集约化上，**启动60个农业示范区建设，全椒襄河、凤阳小岗和来安大英3个示范区获批省级示范区，定远金山示范区获批省级农业综合开发区，全椒争创国家级示范区工作稳步推进。**规模化上，**新增耕地流转面积41万亩，总面积达182万亩，流转率高于全省1.3个百分点；新增家庭农场780家，总数达1500家；新增各类农民合作组织1160个，总数达5860个。**产业化上，**新增各类龙头企业99家，总数达645家；实现农产品加工产值468亿元，增长20.3%。**科技化上，**农业物联网扎实推进，良种覆盖率、关键技术到位率分别达97%和68%。圣农科技、意洋草莓等现代农业项目产生良好示范效应。**机械化上，**耕种收农机化作业水平达78.9%，超全省14个百分点，农机化工作综合考评全省第一。

**（五）突出提档升级，第三产业呈现新格局。**

坚持生产性服务业和生活性服务业并重，现代服务业和传统服务业并举，全年实现服务业增加值308亿元。**商贸服务业更趋活跃。**净增限上企业182户，累计达762户。沃尔玛、大润发等陆续开业，世贸广场、中普广场等加快建设，在建大型市场26个、商业综合体19个。总投资120亿元的中环国际商贸中心项目签约。发展电子商务，年交易额1.5亿元，筹建淘宝特色中国·滁州馆。**现代物流业加速推进。**中国供销·滁州农产品物流园、华塑盐化物流园、凤阳港二期等项目加快建设，完成投资26.5亿元。南京湾商贸物流园获批省级服务业集聚区。**文化旅游业快速提升。**围绕打造中国优秀旅游目的地城市，加快推进文化旅游项目建设。长城文化创意园、滁州1912、冠景度假村、全椒儒林文化街区、天长千秋印象·唐城等项目快速推进。**金融服务业增势强劲。**县域农商（合）行改制全面完成。新增18家银行分支机构，新引进1家市级保险公司。先后与省进出口银行、省建行和徽商银行签订战略合作协议，共获授信650亿元。存、贷款增速分别居全省第七、第二。**房地产业持续增长。**完成商品房建设投资261亿元，增长13.9%；销售472万平方米，增长38.4%，增速均居全省前列。

**（六）突出宜居宜业，城乡面貌发生新变化。**

坚持规模扩张、功能完善、品质提升并举，努力构建多层次城镇体系。**以美好新滁城为龙头，着力提升品质。**“131”组团实施重点项目412个，完成投资296亿元，增长41%。大力实施以“七个一”为重点的精品工程，清流高架、南湖四期等项目竣工，高新技术创业服务中心等项目提速，琅琊山东麓覆绿和醉翁亭景区整治、滁州大道、花卉博览园等项目开工建设。“十路一渠”建设快速推进，花园路、全椒路、会峰路“白改黑”完成，西涧路、洪武路、世纪大道改造等工程加快建设。实施清流河二期、扬子东路、苏滁现代产业园等地块征迁，完成征收面积141万平方米。组团内部竞相发展，琅琊新区建成百万平方米住房，南谯新城一批公共设施项目进展明显，来安县城“一城三区”路网框架基本形成，全椒十大城建、八大交通工程顺利推进。以城市管理十项提升行动为抓手，深入推进“六城联创”，完成上海路、丰乐大道、中都大道提速治堵工程。推进“绿色滁州行动”，新增绿地585万平方米，城区绿化覆盖率达39.4%。**以美好新城镇为支撑，着力完善功能。**天长、明光、凤阳、定远四个县城共实施重点城建项目近300个，完成投资近200亿元。天长建成红草湖南园等30项工程。明光征收房屋面积70万平米，韩山公园二期等15项工程完工。凤阳提升文化新城，博物馆、新图书馆即将投入使用。定远生态公园、滨水商业休闲带等工程进展顺利。炉桥等一批特色小城镇加快建设。全市城镇总人口184万人，城镇化率达46.8%。**以美好乡村为基础，着力示范带动。**按照串珠、连线、带面模式，聚力打造“一片二环三线”，重点抓好5条展示链和38个重点村建设，完成各类村庄建设项目818个。**以基础设施建设为载体，着力改善条件。**滁马高速、宿扬高速等开工建设；徐明高速、蚌淮至宁洛高速连接线、104国道滁汊段等加快建设；312省道滁来路、釜秦线、天铜线和309省道明光段等建成通车。蚌浮段行蓄洪区调整建设工程开工，滁河防洪治理近期工程加快推进，5座中型水库、127座小型水库完成除险加固。全年交通投资126亿元、水利投资15.8亿元、电力投资9亿元。市政公用设施建设完成固定资产投资157.6亿元，总量全省第二。

**（七）突出开源节流，保障发展有了新举措。**

坚持多管齐下筹集资金，节约集约使用资金，务求把有限资金用在刀刃上。**抓开源，**完成税收146亿元，占财政收入的比重为81.6%；争取上级专项转移支付74.3亿元，地方政府债券转贷8亿元。全市金融机构本外币贷款余额突破900亿元；同创公司发行债券8亿元，德力股份再融资5.36亿元，海虹印务、尚善生物在区域股权交易中心挂牌，全市社会融资总量450亿元。**抓节流，**建立预算执行动态监控机制，严控“三公”经费等支出，市直部门一般性支出压减6%，市本级政府采购节约率达30%，收回历年结转资金4025万元。加大政府投资项目审计力度，仅市级核减工程造价2.45亿元。**抓调度，**按照“突出重点、急用先行、有增有减”的原则，调整优化支出结构。市财政全年调度资金76亿元，有效保障重点项目建设及偿还到期债务。

**（八）突出民生福祉，社会事业再获新进展。**

坚持把保障和改善民生摆在突出位置，加大投入，狠抓落实。**民生工程上，**33项民生工程投入54亿元，比上年增加2.4亿元。新开工保障房2.7万套，建成3.8万套，居全省前列。新增城镇就业9.4万人，城镇登记失业率3.5%，低于省控指标1个百分点。城镇职工五项保险参保人数达155万人次；新农合平均参合率首次达100%。**教育上，**完成滁州职教集团组建工作，组建滁州机电工程学校。完成126所义务教育标准化学校建设任务，新建公办幼儿园79所。**文化卫生体育上，**建成6个县级文化馆、图书馆、博物馆，20个村级文化活动中心示范点，49个公共电子阅览室。明中都城遗址被列入国家考古遗址公园立项名单。琅琊山摩崖石刻及碑刻跻身“国保”。县级公立医院改革稳步推进，重点医疗机构加快建设，市中西医结合医院成功创“三甲”。基层医改深入推进，药品价格总体降幅40%左右，医用耗材集中采购中标价下降33.6%。建立医患纠纷调解新机制。5名滁州籍运动员在十二届全运会上夺冠。**人口计生上，**深化“村为主”工作机制，健全计划生育利益导向政策体系，人口自然增长率5.5‰。**信访维稳上，**深化平安滁州建设，推行治安防控四色分级预警机制，实施“天网”工程，新增治安监控6000多个，防控能力得到提升。严厉打击各类违法犯罪，成功破获“10.24”爆炸未遂案等刑事案件。创新信访工作机制，打造“信访直通车”，排查调处各类矛盾纠纷近6万件。突出重点行业领域安全监管，事故起数和死亡人数均下降50%以上，没有发生重特大安全事故。**环境保护上，**加强制度建设，严格环境准入，强化执法监管，节能减排目标任务全面完成，新增4个省级生态乡镇、9个省级生态村。琅琊榆、滁菊被评为市树、市花。做好全市第三次全国经济普查准备工作。《滁州市志》出版发行。老龄、红十字、慈善和残疾人等事业取得新成绩，工会、共青团、妇联、文联、科协等群团组织作用进一步发挥，统计、质监、商检、民族宗教、外事、侨务、工商联、防震减灾、气象、国防动员、消防、保密、档案、编制、供销、老干部、关心下一代等工作全面加强。

**（九）突出勤政廉政，政府自身建设得到新加强。**

坚持依法行政、从严治政、廉洁从政，不断加强和改进政府工作。认真执行市人大及其常委会的决定、决议，主动接受市人大的法律监督、工作监督和市政协的民主监督，广泛听取各方面意见。共办理人大议案、建议120件、政协提案294件，满意率达95%。修改完善政府工作规则。加强政府规范性文件审查，清理文件459件，依法行政水平进一步提高。开展第六轮审批流程再造，推进市县两级公共资源交易市场一体化。完善政府信息公开发布机制，加强市长热线、市长信箱办理工作，积极开展网络问政。全面落实党风廉政建设责任制，严格执行中央八项规定、省30条和市26条规定，认真开展办公用房清理工作，深入推进“六治六提”活动，强化行政监察和审计监督，严肃查处违纪违法案件，反腐倡廉建设进一步加强。

各位代表，过去的一年，我们在“十二五”的征程上迈出了更加坚实有力的步伐。成绩来之不易，这是省委、省政府和市委坚强领导的结果，是市人大、市政协监督支持的结果，是全市人民团结奋斗的结果。在此，我代表市人民政府，向全市人民，向市人大代表、政协委员和党代会代表，向各民主党派、工商联、无党派人士，向各人民团体、部省驻滁单位和离退休老同志，向驻滁人民解放军、武警官兵、公安政法干警，向所有关心、支持、参与滁州建设和发展的海内外朋友们，致以衷心的感谢和崇高的敬意!

在肯定成绩的同时，我们也清醒地看到，滁州的发展还面临诸多困难，政府的工作还存在不少问题，如：地区生产总值、固定资产投资、城乡居民收入等指标增速未能实现年初计划目标；大项目、大企业、大产业的支撑作用还不够明显，经济外向度、全民创业水平、自主创新能力还有待进一步提高；县域经济特别是镇域经济仍较薄弱，发展不平衡现象仍较突出；资源环境承载的压力日渐增大，要素保障的压力一直存在，信访维稳的压力不同程度影响了一些地方和干部的正常工作；改革力度有限，没能有效触及一些体制性的障碍和深层次的矛盾；民生改善与人民群众的新要求、新期盼还有不小差距；政府及所属部门的服务水平、服务效率、服务质量还有待进一步提升，等等。对此，我们将高度重视，下大力气加以解决。

二、2014年工作安排

2014年是贯彻落实十八届三中全会精神、全面深化改革的开局之年，是实施“十二五”规划的攻坚之年，也是我市打造经济升级版的关键之年。尽管我们面临着宏观环境复杂多变与区域竞争日益激烈的双重压力，做大经济总量与加快转型发展的双重考验，发展要素需求不断增多与资源环境约束不断增强的双重矛盾。但对滁州而言，机遇大于挑战，我们正处于大有可为的黄金发展期、转型升级的战略机遇期、深化改革的重要窗口期。多重机遇叠加的好形势仍将延续，多个项目形成的产能将持续释放，多年努力打下的坚实基础将继续发挥其强有力的支撑作用。只要我们咬定目标，锐意进取，攻坚破难，就一定能够实现经济社会更好更快发展。

今年工作的指导思想是：全面贯彻落实党的十八大和十八届三中全会等重要会议精神，以稳中求进为工作总基调，以全面深化改革为根本动力，以转型升级为主攻方向，以争先进位为主要目标，着力激发市场活力，着力提升发展质量，着力加强城乡统筹，着力推动民生改善，加快冲刺全省第一方阵。

经济社会发展主要预期目标是：地区生产总值增长11%左右，固定资产投资增长18%，财政收入增长12%，社会消费品零售总额增长13%，外贸进出口增长11%，城镇居民人均可支配收入、农民人均纯收入分别增长11%和13.5%，居民消费价格涨幅控制在3.5%左右，城镇登记失业率控制在4.4%以内，人口自然增长率控制在6‰以内，完成年度节能减排任务。

**（一）以深化改革为引领，汇聚发展活力。**

按照中央和省统一部署，弘扬大包干精神，做到谋定而后动、在大局下行动，努力以点上的有效突破，迅速打开改革新局面。**深化农村改革。**探索推行地权、林权、房权的流转租赁和抵押融资，培育新型农业经营主体，推进国有林场、承包经营权入股、资金互助、土地流转、小型水利工程建管等改革，推进凤阳县省级农村综合改革示范试点，抓好土地确权登记颁证试点，争取走在前列。**深化企业改革。**认真开展国企改革“回头看”活动，逐项解决好遗留问题。引入非公资本，发展混合所有制经济。加快建立现代企业制度，引导民企通过挂大靠强、兼并重组、股改上市等途径，加速成长。**深化财税体制改革。**建立政府举债审核备案和偿债准备金制度，实行全口径预算管理，做好政府债务风险防控和化解工作；深化财政支出改革，规范支出方式，全面公开“三公”经费，推行预算支出绩效评价；推进税收征管改革，做好“营改增”扩围试点。**深化行政体制改革。**加快政府机构改革和部门职责整合，完成工商、质监管理体制调整和食品药品监管体制改革，完成卫生与人口计生机构整合。加快推进事业单位分类改革，进一步理顺关系和去行政化。**深化城市建设管理体制改革。**加强城市资源的整合和开发，引入市场化、社会化机制，盘活存量，搞活增量。改革、完善滁城的征迁、安置、环卫、物业、社区管理体制机制。实行居住证制度，全面放开县城以下户籍和有序放开滁城户籍登记；统筹解决好进城人员的住房、就业、养老、就医和子女上学等问题，让农民“进得来”、“留得住”。**深化社会领域重点改革。**深入推进文化体制改革，按照政企分开、政事分开的原则，进一步理顺政府与文化企事业单位的关系；统筹城乡文化发展，推进基本公共文化服务均等化。深入推进医药卫生体制改革，巩固完善基层医���，深化推进县级、适时启动市级公立医院改革。完善社会保障体系，推进新农合和城市居民医保并轨。

**（二）以项目建设为抓手，增强发展后劲。**

**强力抓好招商引资。**力争市外亿元以上项目到位资金700亿元以上，增长20%以上。一是突出招大引强。围绕主导产业，主攻重点区域，紧盯大企业、大项目，高位推进，力争新签约亿元以上项目400个。二是创新招商方式。尤其要在围绕产业链组建专业队伍招商和驻点招商、网络招商和以商招商、集群承接和共建园中园等结合上下功夫。积极利用上海自贸区效应，主动把握国家促进产业向中西部转移的政策机遇，加强承接能力建设，提高对外开放水平。加速融入南京都市圈和合肥经济圈，左右逢源、借力发展。三是注重招商实效。完善考评机制，重点考核项目亩均投资强度、产出和税收等指标，严控高耗能、高污染、低效益项目落地；强化对重点项目的调度、督查，提高项目签约率、开工率、竣工率、达产率、履约率、贡献率。

**全力推进项目建设。**一方面抢抓机遇谋项目。加强对国家、省投资导向的研究，抓好项目谋划、申报、衔接等工作，争取一批事关长远发展的项目落户滁州。加速推进淮滁宁高速、合明盱高速、通用机场、窑河复航、海关特殊监管区等项目，力争年内大唐电厂项目申报核准，江巷水库项目获批，滁州港、城东热电厂、50万伏变电站等项目开工，滁宁轻轨、合宁城际铁路等前期工作取得突破。用好用足皖北扶持政策，促进定、凤、明发展。另一方面提质提效建项目。排出年度重点工程，强力推进，全年新开工亿元以上项目240个，竣工投产150个以上，达产100个以上。

**大力推动园区发展。**按照“产城一体”理念，推进各开发园区拓展空间、优化布局，确保15个省级以上园区完成年度建设投资100亿元以上，新增开发面积20平方公里以上，税收收入超65亿元。加快产业集聚区建设，着力打造沿苏经济带。推进苏滁现代产业园争创省级高新区，力争签约引进项目50个，完成固定资产投资30亿元，10个以上项目年底投产。推进市开发区在全国创品牌、全省进位次，主要经济指标增长30%以上。争取天长开发区获批国家级开发区。

**着力强化要素保障。**一是搭建集约的用地平台。一手抓指标争取，力争新增用地指标3.9万亩；一手抓节约用地，把好项目用地审批关，鼓励建设多层厂房，通过土地使用税等杠杆，加大闲置、低效土地的清理力度，确保重点项目用地需求。二是搭建多元的融资平台。抓信贷投放，力争市内银行新增贷款超200亿元。抓直接融资，力争禾益化学、扬子地板“新三板”挂牌，凤凰集团、志成畜牧等在区域性股权交易市场挂牌，市城投三期债、同创二期债、金禾实业公司债、滁宁公司债、金瑞投资短期融资券等成功发行。组织设立民营银行。力争全年社会融资总量超500亿元。三是搭建优质的聚才平台。加大人才和技术引进力度，加强与科研院所、大专院校的合作，引进新技术、新工艺、新成果100项。办好学子家园网、农民工家园网，助推企业招工。四是搭建高效的服务平台。深化“三个一千”行动，发挥“四大平台”作用，不断提升帮招、帮抓、帮扶水平。

**（三）以转型升级为主线，提升发展质量。**

**在育大育强中推进工业转型升级。**牢固树立工业立市、工业强市理念，着力培育市、县两级各自的主导产业及首位产业。一是壮大主导产业促转型。积极推进“十百千”支撑工程，深入开展“千企升级”、“千亿技改”行动，加大工业投入，力争全年完成工业固定资产投资650亿元，其中技改投资300亿元。全力支持安康电子、博西华、天大、全柴、天康、蓝德、中普能源、金禾实业等做大做强，力争康佳率先过百亿；新增规模以上工业企业150户以上。二是发展新兴产业促转型。大力发展信息、光电、新材料、生物制药和节能环保产业。加大对战略性新兴产业的投入和政策支持，高新技术产业产值占比达40%以上。三是推进“两化融合”促转型。推进信息化和工业化融合发展，完成工业企业主辅分离10家以上，两化融合示范区增加到4个，省级两化融合企业增加到30户以上，新增智慧企业150户以上。四是培育民营经济促转型。落实扶持民营经济发展优惠政策，消除隐性壁垒，培育创业主体，推动全民创业。力争新增私营企业超5000户、个体工商户超2万户，民营经济占GDP比重达64%。

**在又好又快中推进服务业转型升级。**以实施大市场、大物流和大旅游工程为突破口，推进服务业发展提速、比重提高、水平提升。一是大力发展现代物流业。围绕长江北岸区域性物流中心和粤赣皖苏物流大通道重要节点城市，实施物流基地工程，加快建设高铁站区低碳服务业集聚区、京沪铁路物流集聚区和滁州港综合物流园等一批物流中心，新培育1家4A级物流企业。二是优化发展现代商贸业。加快中环国际商贸中心、亚太五金机电城、苏宁广场等项目建设，争取意大利名品城、红星美凯龙、义乌商贸城等项目尽早开业。实施电子商务突破工程，引导现有企业加快“触电”，争创国家电子商务示范城市和示范基地。三是提升发展文化旅游业。争取“中国农民歌会”这一品牌项目获国家批准。加快发展文化产业，积极引进一批文化创意、动漫制作等项目。快速推进长城文化创意园、森禾花博园、77创意园区，以及南谯远望东基、全椒神山和龙山、明光跃龙湖和栖凤湖、来安舞彩、天长金牛湖等旅游项目建设。围绕琅琊山旅游区“两年大变样”目标，加快醉翁亭景区、冠景度假区、中华牌匾园、东方醉园等项目建设，争创5A级景区。小岗村旅游区争创5A级景区，凤阳韭山洞和明皇陵、天长红草湖等争创4A级景区。四是健康发展房地产业。出台政策措施，活跃房地产市场，鼓励高校毕业生、进城务工人员和外地人口买房；加大住房有效供给，改善居民居住条件，促进房地产业健康发展。

**在做优做特中推进农业转型升级。**以增加农民收入为核心，以工业理念推动现代农业园区建设，让农业成为有奔头的产业，让农民成为体面的职业，让农村成为安居乐业的美丽家园。抓好示范化引导。加快全市81个现代农业示范区建设，力争全椒创建国家级示范区取得成功，南谯、明光、定远三地创建省级示范区取得突破。创建50个市级示范家庭农场、20个市级示范合作社。扩大规模化生产。新增土地流转30万亩，新培育发展家庭农场500个，新发展农民合作组织1000个。推进产业化经营。重点扶持美国嘉吉、雀巢银鹭、养元饮品、盼盼食品、德青源、舜田科技等龙头企业发展。新增各类龙头企业超百家，农产品加工产值达520亿元。加快品牌化发展。加大无公害农产品、绿色食品、有机食品认证力度，注册一批特色商标，争创一批知名品牌，增强农业竞争力。夯实设施化基础。推进88万亩高标准农田建设，完成5座中型水库、74座小型水库除险加固，加快蚌浮段行蓄洪区调整建设、滁河和池河防洪治理、小农水重点县、大中型灌区等项目建设。建设好强对流天气监测预警系统。

**在创新创造中保障产业转型升级。**突出企业主体。加大高新技术和创新型企业培育力度，力争新认定国家级高新技术企业30家、省市级创新型（试点）企业20家。全面推进亿元以上企业建立技术中心或研发中心。突出创新载体。推进南京工业大学科教城项目，更好建设汽车与家电技术及装备研究院、中国质量认证中心滁州分中心等公共创新平台，支持全柴、天康、扬子空调、鲲鹏模具等创建国家级工程研究中心，新组建院士工作站1家、博士后流动站2家，新建定远、明光、凤阳、琅琊4家创新服务站，实现“科技路路通”县级服务站全覆盖。突出产学研一体。积极寻求与高校院所以及学科领头人、专业技术团队合作办园区、办企业，组织开展各类产学研活动30场次，每个省级以上开发区达成合作项目10个以上，实施项目5个以上。

**（四）以城乡统筹为路径，建设美好家园。**

走科学发展的新型城镇化道路，更加注重规划引领，更加注重以人为本，更加注重产城融合，更加注重生态文化，更加注重城乡统筹，更加注重集约建设，努力“让人生活更美好”。

**提升滁城牵动力。**大滁城“131”组团实施重点项目360个，完成投资220亿元。一是提升品质化。围绕“七个一”重点工程，开工建设清流河二期工程和龙池街改造项目，启动明湖区域房屋征收和蓄水工程，推进儒林湖景区建设和古城周边环境整治。城南新区四大城市综合体建成，港汇喜来登酒店、凯迪温德姆酒店运营；琅琊新区和南谯新城加快推进道路、安置房、公共设施等政府性投资项目建设，展现城市新貌。二是突出功能化。完成城市职业学院新校区一期建设，实现市二院整体搬迁，加强城南学校、菜场、公交、公园等设施配套，加快电力、燃气、通信、供排水等管网系统和“五小工程”建设，编制城市天际线、色彩、雕塑、慢行系统等规划，建设城市亮化系统和公共自行车系统。深入开展“六城联创”，实施十大专项行动，开展“三小车”综合整治，提升城市管理水平。三是加快同城化。对外推进滁宁同城化，抓好104国道改扩建、滁宁轻轨、滁河航道提升等工程。对内推进“131”组团一体化，重点建设滁州大道、洪武大道、宁滁大道、苏滁大道、世纪大道、西涧路等骨干路网，使各组团快连快通。全椒县城北扩、来安县城南进，实现与滁城的无缝对接；汊河新城实现“两年大变样”目标。

**增强城镇集聚力。**依托区位交通、特色���业、历史文化和旅游资源，提升城镇文化品位，强化城镇产业支撑，彰显城镇个性魅力。天长、明光、定远、凤阳四个县城要继续繁荣发展新城区，改造提升老城区，新建续建项目近400个，完成投资超300亿元。天长启动南市区6平方公里组团开发和东市区4平方公里路网建设，推进老城改造。明光揽山引湖入城，着力推进东部、北部新城建设。定远加快推进体育中心等项目建设，改造、提升县城各出入口，引导城市向泉坞山、曲阳湖方向发展。凤阳要在完善新城区的同时，把分块改造老城区摆上重要位置，积极规划建设府城经临淮至凤宁开发区的发展轴。同时，打造一批综合发展型、工业主导型、生态旅游型、商贸物流型、现代农业型城镇，发挥引领作用。

**强化乡村支撑力。**加快实施美好乡村“1313”空间布局，重点建设48个中心村，精致做美10条示范线和4大精品区块，全面整治100个沿线沿边村，完成农村危房改造5800户，实施好农村清洁工程，配套建设垃圾收集和转运设施，实现“点上出彩、沿线美丽、面上整洁、民富村美”。

**提高基础设施承载力。**确保徐明高速建成通车；蚌淮至宁洛高速连接线一期工程建成通车，二期工程主体完工；滁马高速开展路面工程建设；确保滁淮高速上马，力争合宁高速扩建开工；推进滁天高速公路前期工作；继续提升14条普通国省干线公路升级改造，新建500公里村级公路贯通工程，完成交通投资200亿元。加速电网建设，完成电力投资9亿元。加快4G网络建设，基本实现全覆盖，完成通信投资6.6亿元。

**（五）以环境保护为重点，打造生态文明。**

大力推进绿色、循环、低碳发展，让居民“望得见山、看得见水、记得住乡愁”。**全力推进“三线三边”整治。**围绕“四治理一提升”，从治理脏、乱、差入手，大力开展治乱洁化、栽树绿化、提升美化和维护常态化工作。把“三线三边”整治与城镇建设、美好乡村建设、土地和矿山整治、水利兴修、现代农业发展等有机融合起来，实现两年大变样。**大力实施森林增长工程。**点、线、面整体推进，着力建设一批特色林、特色园和森林长廊示范线，完成新增造林20万亩。扎实推进“绿色滁州行动”，启动和实施绿化项目94个，新增绿地564万平方米。**强力推进节能减排。**发展循环经济，节约集约利用土地、水、能源等资源。加快推进污水处理厂、水泥厂等重点减排项目，加快淘汰落后产能。**着力加强环境监管。**严格执行新上项目环评、能评和“三同时”制度，严控重点环境风险源和敏感区域，对化工、重金属、危险废物处理等企业，实行最严格的环境监管，最大限度降低环境安全风险。**努力改善生态环境。**加强滁城西部丘陵山区特别是饮用水源区生态保护。争创2个省级生态乡镇、4个省级生态村。加强对大气污染的监控及综合防治，建立责任体系，落实目标任务，让市民享有更多的蓝天白云、绿水青山。

**（六）以改善民生为根本，增进群众福祉。**

按照保基本、兜底线、促公平、可持续的要求，全面做好改善民生工作。

**抓好发展型民生。**落实就业创业各项扶持举措，做好高校毕业生、农村转移劳动力、城镇就业困难人员的就业工作，扎实推进居民收入倍增计划。加强创业型城市建设，继续推进大学生创业孵化基地、农民工创业园、创业中心等建设，以创业带就业。全年新增城镇就业5.5万人。

**抓好保障型民生。**加快棚户区、老旧小区改造，新开工保障性住房1.9万套。稳步提高城镇居民医疗保险待遇，扎实推进城乡居民养老保险，逐步扩大失业保险基金支出范围。推进“金保工程”二期建设，完善各项社会保险制度衔接与跨区域转移接续。加快发展养老服务业，促进居家养老、社区养老、社会养老协调发展。加强和改进低保工作，努力使困难群众生存有尊严、生计有保障、生活有盼头。

**抓好普惠型民生。**全面完成民生工程年度任务。优先发展教育事业，积极开展多种形式的合作办学，规划新建33所公办幼儿园，完成114所义务教育学校标准化建设任务。加快省级职业教育改革试验区和职教大市建设，推进城市职业学院、机电工程学校新校区建设。支持安徽科技学院、滁州学院发展，推进职业技术学院省级示范性高职院校建设。引进建设国际学校。大力发展文化事业，争取市文化馆开馆、博物馆布展、图书馆开工，打造一批农民文化乐园和社区文体中心。深入开展群众性体育活动，积极备战省十三运。加快发展卫生事业，为群众提供安全有效方便价廉的医疗服务。完善利益导向政策体系，落实好“单独两孩”政策，推动人口计生工作转型发展。争创全国双拥模范城“五连冠”。做好第三次全国经济普查。同时，抓好外事、侨务、对台、宗教、统计、物价、编制、档案、保密、人防、气象、地震、科普、地方志等工作，支持工会、共青团、妇联、科协、工商联、红十字会等工作，发展妇女、儿童、老年人、残疾人等各项事业。

**（七）以促进和谐为目标，创新社会治理。**

坚持源头治理、系统治理、综合治理、依法治理，营造更加和谐稳定的社会环境。**一是加强宣传教育。**推动传统媒体和新兴媒体融合发展，统筹抓好内、外宣工作，宣扬主流思想、树立新形象、传播正能量；深入推进精神文明创建活动，大力实施公民道德建设工程，唱响实现“中国梦”的滁州最强音。**二是抓好信访维稳。**健全信访工作“乡为主”机制，推广“枫桥经验”、“南陵经验”，畅通群众诉求表达、利益协调、权益保障渠道。健全社会稳定风险评估和“大调解”机制，强化法律援助和服务，有效预防和化解社会矛盾。**三是强化安全生产。**强化红线意识，按照“全覆盖、零容忍、严执法、重实效”的要求，认真做好非煤矿山、易燃易爆、交通运输、消防安全等工作，有效防范和遏制重特大事故发生，促进安全生产形势持续稳定好转。整顿规范市场秩序，用最严格的监管、最严厉的处罚、最严肃的问责，确保群众“舌尖上的安全”。**四是深化平安建设。**深入推进“崇尚法治、共创平安”活动，推行“六六联创”，健全治安防控“六张网”，严厉打击各类刑事犯罪，完善社区矫正制度，提升群众安全感和满意度。

三、着力打造人民满意政府

打铁还需自身硬。要以踏石留印、抓铁有痕的劲头加强自身建设、推进各项工作，切实做到善始善终、善做善成，以实际行动，建设人民满意政府。

**强化学习意识，增强行政能力。**政府系统全体人员要牢固树立终身学习的理念，自觉把学习作为一种追求、一种爱好、一种健康的生活方式。切实加强理论、政策、法规和科技、文化知识的学习，特别要深入学习党的十八大、十八届三中全会及习近平总书记系列重要讲话精神，不断提高自身的政治素质、理论素养和政策水平。强化问题意识，带着问题学习，在学习中思考、解决问题，学以致用、用以促学、学用相长。既要精通业务知识，更要提高实干能力；既要熟悉微观情况，更要善于把握宏观走势，力求把学习成果转化为谋划工作的思路、深化改革的举措、推进发展的本领。

**强化宗旨意识，提升服务水平。**全面落实中央、省关于改进工作作风、密切联系群众规定，切实加强政府系统作风建设，尽心竭力履职，奋发有为工作，脚踏实地干事。认真开展以“为民、务实、清廉”为主要内容的党的群众路线教育实践活动，走基层、听民声、接地气，把握民生脉动，关注民生诉求，解决民生困难。加快转变政府职能，落实好中央和省取消、下放的审批事项，公开审批事项，再造审批流程，缩短审批时限，做到“大厅之外无审批、大厅之内快审批”，用政府权力的“减法”换取市场活力的“加法”。

**强化制度意识，坚持依法行政。**自觉接受市人大及其常委会的监督，认真落实市人大及其常委会的决议、决定，定期报告工作。大力支持市政协履行职能，广泛听取各民主党派、工商联、无党派人士和各人民团体意见。认真办理人大代表建议、议案和政协委员提案。深入开展“六五”普法和法治创建活动。坚持依法决策、民主决策、科学决策，对重大决策实行民意调查、合法性审查、专家论证、风险评估、公示听证和集体讨论决定。加强规范性文件管理，严格行政执法程序、执法标准和行为规范。加强对权力运行的制约和监督，把权力关进制度的笼子里。深入推进政务公开、政府信息公开，促进政府工作公开透明，让权力在阳光下运行。

**强化责任意识，创新工作效率。**认真践行习总书记关于好干部“信念坚定、为民服务、勤政务实、敢于担当、清正廉洁”的要求，牢固树立进取意识、机遇意识、责任意识，强化目标管理，营造一种善于决策、敢于负责，不畏艰难、不避矛盾，求真务实、真抓实干，雷厉风行、一抓到底的良好风气。建立健全各项工作机制，不断推进治理体系和治理能力的现代化建设。继续开展“治庸提能、治懒增效、治散聚力、治慢促快、治乱正气、治奢倡俭”活动。推进电子政务，减少会议、文件和迎来送往，提高办事效率。制定政府绩效管理办法，建立科学有效的绩效考评体系，加强过程监控，注重结果问效，提高工作执行力。

**强化廉政意识，保持清廉形象。**严格执行《党政机关厉行节约反对浪费条例》、《党政机关国内公务接待管理规定》等规章制度，厉行勤俭节约，持之以恒反对“四风”。健全严格的财务预算、核准和审计制度，严控“三公经费”支出和楼堂馆所建设。加快推进电子化招投标，完善标前、标中、标后监管。落实党风廉政建设责任制，加强行政监察、审计监督。全面推进惩治和预防腐败体系建设，加大对腐败案件的查处力度，以反腐倡廉的实际成效取信于民、造福于民，努力做到干部清正、政府清廉、政治清明。

各位代表，又是一年春光近。滁州乘势而上的历史机遇在等待着我们，跨越追赶的神圣使命在激励着我们，人民群众对美好生活的殷切期盼在鼓舞着我们，让我们紧密团结在以习近平同志为总书记的党中央周围，在省委、省政府和市委的坚强领导下，深化改革，开拓创新，扎实苦干，拼博奋进，为打造滁州经济升级版、努力贡献中国梦而奋斗！
